# Landscape of targets within nucleoside metabolism for the modification of immune responses

**DOI:** 10.3389/fonc.2025.1483769

**Published:** 2025-05-30

**Authors:** Ella M. Dunderdale, Evan R. Abt

**Affiliations:** Department of Molecular and Medical Pharmacology, University of California Los Angeles, Los Angeles, CA, United States

**Keywords:** metabolism, immune activation, cancer immunotherapy, nucleotide metabolism, autoimmune disease, immuno-metabolism

## Abstract

Nucleoside metabolism regulates immune cell development and function, but the therapeutic implications of this link have yet to be fully realized. Evidence for the importance of nucleoside metabolism in immune system control was provided by observations of immunodeficiency and autoimmunity across patients with genetic errors that alter nucleoside synthesis or breakdown. Research over the past several decades has uncovered a multifaceted role for nucleosides in mediating immune responses that involves their function as metabolic precursors and as ligands for immune receptors. These findings prompted the development of treatments that block the production of the immunosuppressive nucleoside adenosine for cancer immunotherapy. Guanosine and pyrimidine nucleosides also mediate immune outcomes, and the key regulators of their metabolism are promising new targets to unleash anti-cancer immune responses or dampen autoimmune reactions. This review provides an overview of (i) recent research concerning the mechanisms underlying nucleoside-mediated immune regulation, (ii) the current landscape of therapeutic targets for immune modulation within nucleoside metabolism, and (iii) opportunities for developing improved preclinical models that recapitulate human nucleoside metabolism, which are needed to advance new metabolism-targeting therapies toward the clinic.

## 1 Introduction

Nucleosides critically regulate immune system function and targeting nucleoside metabolism has emerged as a promising approach to unleash anti-cancer immune responses or restrain autoimmune reactions. Nucleosides have a multifaceted role in immune system regulation that involves their function as metabolic precursors and signaling modifiers. Nucleosides are classical biosynthesis metabolites that fuel nucleotide production and nucleic acid synthesis. Nucleosides are also signaling molecules that regulate biological outcomes by engaging intracellular or cell surface-localized receptors. The immunosuppressive properties of the purine nucleoside adenosine are well-studied, and therapies that block adenosine production are currently under clinical investigation for cancer immunotherapy (1).

Recent research has revealed that nucleosides beyond adenosine also mediate immune-related outcomes. These nucleosides include (deoxy)guanosine and the pyrimidine nucleosides (deoxy)cytidine, uridine, and thymidine. The mechanisms underlying the immune-modifying properties of these metabolites are not as well-studied as adenosine, and therapeutic strategies to leverage their immune-modifying properties have not yet been systematically tested in the clinic. There has been a disproportionate focus on adenosine over pyrimidine or other purine nucleosides in the context of research related to immune system regulation. The striking manifestations of immune dysfunction in patients with diminished activity of the adenosine metabolizing enzyme adenosine deaminase (ADA), first described in the 1970s, may have contributed to this discrepancy. However, the proteins controlling pyrimidine or guanosine nucleoside metabolism may be equally crucial therapeutic targets to modify immune outcomes as those controlling adenosine-mediated immunosuppression.

New studies have highlighted the potential for targeting key regulators of guanosine and pyrimidine nucleoside synthesis, utilization, or breakdown to amplify immune responses against cancer or dampen autoimmune reactions. However, there is an incomplete understanding of the molecular mechanisms underlying the immune-regulatory properties of nucleosides beyond adenosine. This gap in knowledge may be addressed through future studies in improved preclinical models and the analysis of specimens from ongoing clinical trials testing inhibitors of adenosine metabolism for cancer treatment.

A challenge in the development of metabolism-targeting drugs for immune modification is the paucity of preclinical models that recapitulate human nucleoside metabolism. Significant differences exist in the nucleotide metabolism of humans and conventional laboratory models such as rodents. For example, the pyrimidine nucleosides deoxycytidine and thymidine are measured at a 100-fold higher concentration in murine sera compared to sera from humans or non-human primates (2). This discrepancy presents a major obstacle to implementing the findings from laboratory investigations in the design of clinical trials (3). Also contributing to the challenge of translating preclinical research findings are differences in the expression patterns of immune sensor proteins across humans and mouse models. Research using preclinical models that recapitulate both human nucleoside metabolism and immune responses may provide the insight needed to advance new therapies to alter immune-related outcomes in patients.

The goals of this review are to (i) highlight primary research articles that have demonstrated functions of nucleosides beyond adenosine in immune system regulation, (ii) provide an update on recent advances in targeting nucleoside metabolism for cancer immunotherapy, and (iii) summarize the challenges and opportunities related to the development of preclinical models for human nucleoside metabolism that are needed to advance new metabolism-targeting therapies toward the clinic.

## 2 Immune-regulatory functions of guanosine nucleosides

Evidence for the immune-regulatory roles of nucleosides was provided by the identification of immune dysfunction in patients with hereditary loss-of-function mutations in two genes responsible for the breakdown of purine nucleosides: ADA and purine nucleoside phosphorylase (PNP) (4, 5). A leader of these investigations was the physician-scientist Eloise Giblett (6), whose interest in purine metabolism began when she identified a complete lack of blood ADA activity in a patient with severe combined immunodeficiency (SCID). ADA is an enzyme within the purine salvage pathway that catalyzes the deamination of adenosine and deoxyadenosine nucleosides to inosine or deoxyinosine, respectively. Giblett and colleagues also identified that the mutational inactivation of PNP, an enzyme that is also involved in purine metabolism, produces a near-complete absence of T cells alongside altered phenotypes of other immune lineages (5). These foundational studies that associated defects in purine metabolism with the development of SCID provided compelling evidence for a role of nucleoside metabolism in regulating immune responses.

SCID is an established manifestation of PNP/ADA deficiency or defects in other genes that control immune responses. It is rare across the human population, occurring in 0.001-0.002% of births. ADA deficiency constitutes 10-15% of SCID cases (7). Enzyme replacement, hematopoietic stem cell transplantation (HSCT), and gene therapies enable the management of ADA-linked SCID (8). In contrast, only a very small fraction of SCID cases are due to defects in PNP; approximately 70 cases of PNP deficiency have been documented (9). The T cell deficiency associated with PNP inactivation is managed in the clinic with HSCT alongside other treatments.

PNP is a key regulator of the purine salvage pathway. PNP catalyzes the release of purine nucleobases that can be recycled by hypoxanthine phosphoribosyltransferase (HPRT), an enzyme which conjugates nucleobases with phosphoribosyl pyrophosphate (PRPP) to generate purine nucleotide monophosphate (10). In addition to enabling the intracellular purine salvage pathway, PNP controls the systemic levels of purine nucleosides. PNP deficiency results in the systemic accumulation of guanosine, adenosine, inosine, deoxyguanosine (dG), and deoxyadenosine (dA). PNP is also critical for the conversion of purine nucleosides to uric acid and their subsequent excretion (10).

The observations of T cell deficiency in PNP-deficient patients made by Giblett and colleagues have been confirmed by other groups who have expanded the catalog of altered immune phenotypes associated with PNP deficiency in humans (11). A subset of patients with PNP deficiency exhibit autoimmune phenotypes that include systemic lupus, autoimmune hemolytic anemia, and systemic juvenile idiopathic arthritis with macrophage activation syndrome (12, 13). Across patients with PNP deficiency, recurring PNP mutations have been identified that lead to immune dysfunction and susceptibility to infections (14). Partial PNP deficiency is associated with milder symptoms than complete inhibition, and patients with partial PNP activity can exhibit typical development and potentially near-normal immune activity (15).

**Figure 1:**
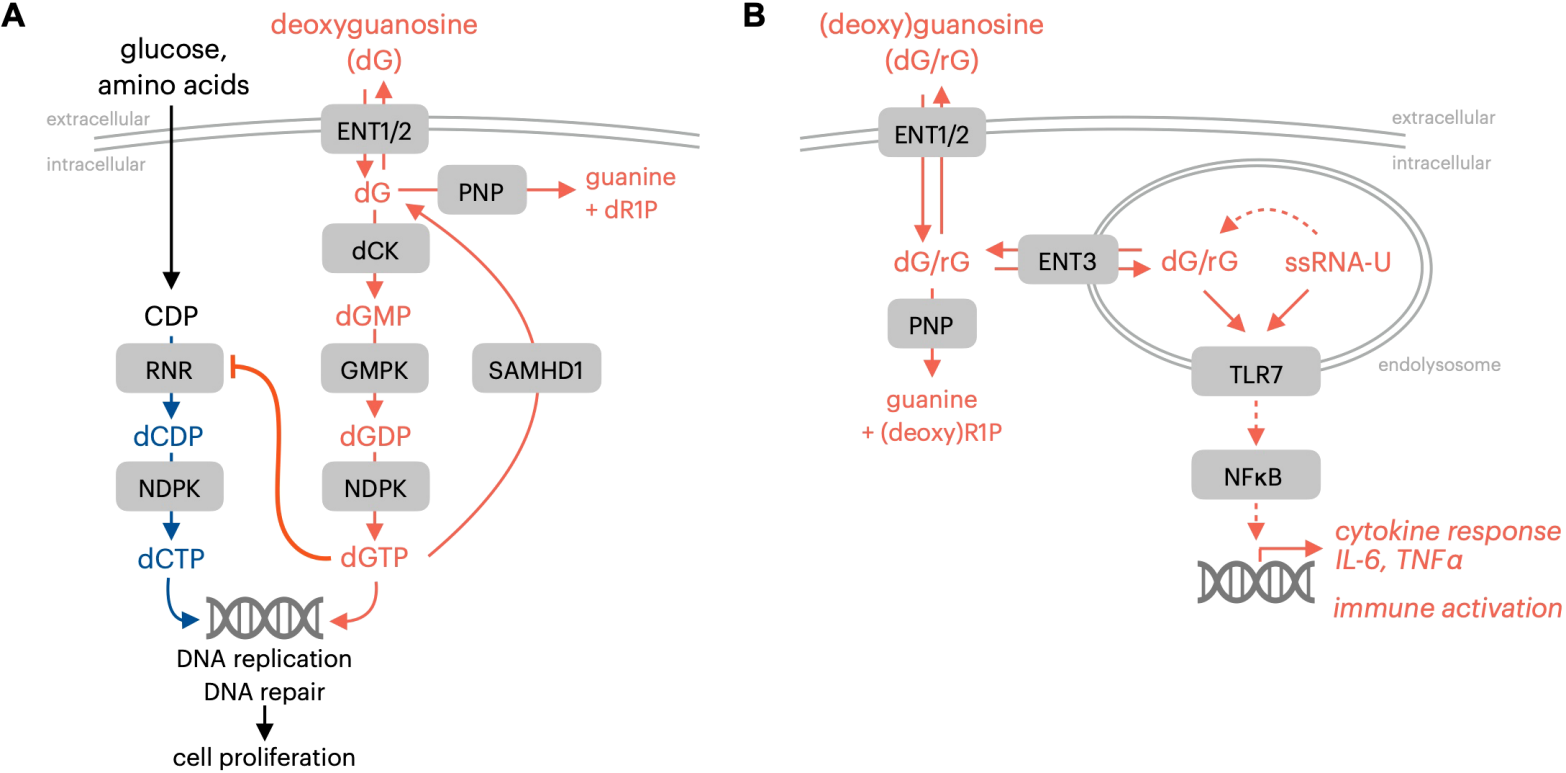
Immune-regulatory effects of guanosine nucleosides. **(A)** PNP and SAMHD1 prevent dGTP-mediated proliferation inhibition resulting from impaired dCTP synthesis. **(B)** PNP limits TLR7 activation by initiating guanosine nucleoside breakdown. RNR, ribonucleotide reductase; NDPK, nucleotide diphosphate kinase; ENT1/2, equilibrative nucleoside transporter 1/2 (SLC29A1/2); dCK, deoxycytidine kinase; GMPK, guanosine monophosphate kinase; SAMHD1, SAM and HD domain-containing protein 1; PNP, purine nucleoside phosphorylase; ENT3, equilibrative nucleoside transporter 3 (SLC29A3); TLR7, toll-like receptor 7; ssRNA-U, uridine-containing single-stranded RNA; dR1P, deoxyribose-1-phosphate; rG, guanosine; dG, deoxyguanosine.

The most profound phenotype observed in PNP-deficient patients, a near complete T cell immunodeficiency, is linked to the uncontrolled expansion of purine nucleotide pools in developing thymocytes following PNP inactivation. The accumulation of the PNP substrate deoxyguanosine and its subsequent metabolism in cells results in dNTP pool imbalance, DNA replication defects, and cell death. The stabilization of deoxyguanosine following PNP inhibition results in a massive expansion of the deoxyguanosine triphosphate (dGTP) pool in cells, which inhibits pyrimidine dNTP synthesis via ribonucleotide reductase (RNR) by an allosteric regulatory mechanism (**Figure 1A**) (16). The entry of deoxyguanosine nucleosides into cells and their subsequent phosphorylation to dGMP is mediated by the sequential activity of transmembrane nucleoside transporters and deoxycytidine kinase (dCK) (17). The preferred dCK substrate is dC. However, dCK also catalyzes the phosphorylation of the purine deoxyribonucleosides deoxyadenosine and deoxyguanosine to dAMP and dGMP, respectively (18). While dCK activity is suppressed via dCTP-mediated allosteric regulation, it is not susceptible to feedback regulation by purine deoxyribonucleotides. Therefore, additional mechanisms must function to counteract purine dNTP pool expansion in the context of PNP deficiency. Developing thymocytes are particularly vulnerable to PNP inactivation due to limited dNTP catabolism capacity, which exacerbates intracellular dGTP accumulation (10). The dNTP triphosphohydrolase SAM domain and HD domain-containing protein 1 (SAMHD1) is expressed at low levels across the early stages of thymocyte development, and this may explain the increased sensitivity of this lineage to PNP inhibition and the resulting uncontrolled expansion of dGTP pools (10). PNP inactivation is synthetically lethal with downregulation of SAMHD1, and this collateral dependency extends to SAMHD1-deficient cells from multiple lineages beyond lymphocytes (10). The transcriptional down-regulation of SAMHD1 during T cell development may be related to the increased dNTP demands of proliferating thymocytes for DNA replication, or the direct role of SAMHD1 in DNA repair by promoting homologous recombination (19). The lethal effects of purine dNTP imbalance are also apparent in T lymphoblastic leukemia cells with high levels of dCK expression alongside low levels of SAMHD1 expression (10).

The autoimmune manifestations related to PNP deficiency in humans are not explained solely by the cell-autonomous lethality that results from the intracellular expansion of purine dNTP pools and the resulting impairment of pyrimidine nucleotide synthesis. Recent research indicates that autoimmune consequences of PNP deficiency may be linked to the sensing of PNP substrates guanosine and deoxyguanosine by the endolysosomal pattern recognition receptor toll-like receptor 7 (TLR7; **Figure 1B**) (20). The toll-like receptor (TLR) family of proteins is a membrane-bound subset of pattern-recognition receptors that are responsible for sensing pathogens and initiating protective immune responses (21). TLRs are vital mediators of innate immune responses that detect pathogen-associated molecular patterns (PAMPs) and subsequently trigger a signaling cascade to activate cytokine production and stimulate immune responses (22). Endolysosomal TLR7 possesses two ligand binding sites that recognize either guanosine nucleosides or single-stranded uridine-containing ssRNA. Gain-of-function TLR7 mutations that result in enhanced guanosine sensing have been identified in patients with early onset systematic lupus erythematosus (23). This observation provided functional evidence for the role of guanosine nucleosides in regulating TLR7 activity in humans (23). Engineered mouse models with TLR7 mutations that increase its binding affinity for guanosine exhibit altered B and T cell function alongside autoimmune manifestations that recapitulate the clinical observations (23).

The effects of acute PNP inhibition on TLR7-mediated immune responses have been reported by multiple groups who have characterized immunological alterations in preclinical models triggered by PNP inhibitors (10, 24). The elevated systemic levels of guanosine nucleosides resulting from pharmacological PNP inactivation impact the function, proliferation, or survival of specific immune lineages as a function of their expression of SAMHD1, dCK, and TLR7. PNP inactivation promotes the expansion of germinal center B cells and populations of T follicular helper cells within secondary lymphoid tissues in the absence of exogenous antigen (10). PNP inhibitor-stabilized guanosine also triggers the production of inflammatory cytokines, such as IL-6 and TNFα, within TLR7-expressing macrophage populations when administered alongside single-stranded uridine-containing RNA (10).

A third manifestation of PNP deficiency is neurological alterations. Preclinical evidence highlights the critical role of PNP activity in neuron survival. The differentiation of induced pluripotent stem cells (iPSC) from PNP-deficient patients provided a platform to investigate the role of PNP in neurons (25). PNP deficiency is associated with enhanced p53-dependent intrinsic apoptosis in this setting, and RNR dysfunction was implicated as a mechanism underlying this effect. PNP also enables the utilization of inosine as a fuel for the pentose phosphate pathway, which has been implicated in the control of neuron function (26). In patients with partial PNP activity, neurological development was found to be normal (15).

Neurological manifestations are also produced by genetic defects in HPRT, a gene that functions downstream of PNP in the purine salvage pathway. Lesch-Nyhan syndrome, caused by the near-total impairment of HPRT, disrupts the synthesis of GMP and IMP nucleotides from guanine and hypoxanthine via the purine salvage pathway (27). HPRT deficiency blocks purine salvage, but spurs increased *de novo* pathway synthesis (27). Diminished guanosine salvage may alter the function of GTP-based secondary messenger systems operating in the central nervous system (27). The guanosine nucleotide GTP, which can be produced via the salvage of PNP products, functions in developmental neurology and controls cell migration, dendrite formation, and neurite outgrowth (28). Guanosine is also a regulator of glutamate re-uptake in glial cells. Therefore, altered guanosine metabolism could impact glutamatergic signaling (29). The phenotypes associated with PNP deficiency and HPRT deficiency reinforce the critical role of purine nucleoside salvage in a neurological context.

In summary, preclinical and clinical studies have pinpointed the role of the nucleoside PNP substrates as critical regulators of immune system function. These effects of guanosine (deoxy)ribonucleosides are related to their roles as (i) ligands for the intracellular pattern recognition receptor TLR7, (ii) substrates for dCK and subsequently fuel for dGTP synthesis, and (iii) substrates for the purine salvage pathway mediated by the sequential actions of PNP and HPRT.

## 3 Immune-regulatory functions of pyrimidine nucleosides

Recent preclinical studies have provided insights into the mechanisms underlying the immune-regulatory functions of pyrimidine nucleosides. Similar to guanosine, the function of pyrimidine nucleosides in immune system regulation involves their roles as metabolic precursors and as TLR ligands. Pyrimidine ribonucleotides, such as (deoxy)cytidine, uridine, and thymidine, can be produced by convergent *de novo* and salvage pathways in cells, and this redundancy allows for metabolic plasticity and adaption to alterations in the availability of environmental nutrients (30, 31). The *de novo* pathway utilizes glucose, glutamine, and aspartate precursors in a six-step biochemical process to produce pyrimidine nucleotides (32). An alternative salvage metabolic pathway for pyrimidine nucleotide synthesis utilizes preformed nucleosides and deoxyribonucleosides (dN) from the extracellular environment. Nucleotide synthesis via the salvage pathway requires the transport of pyrimidine nucleosides across the plasma membrane by nucleoside transporter proteins and their subsequent phosphorylation by intracellular nucleoside kinases (33, 34).

**Figure 2:**
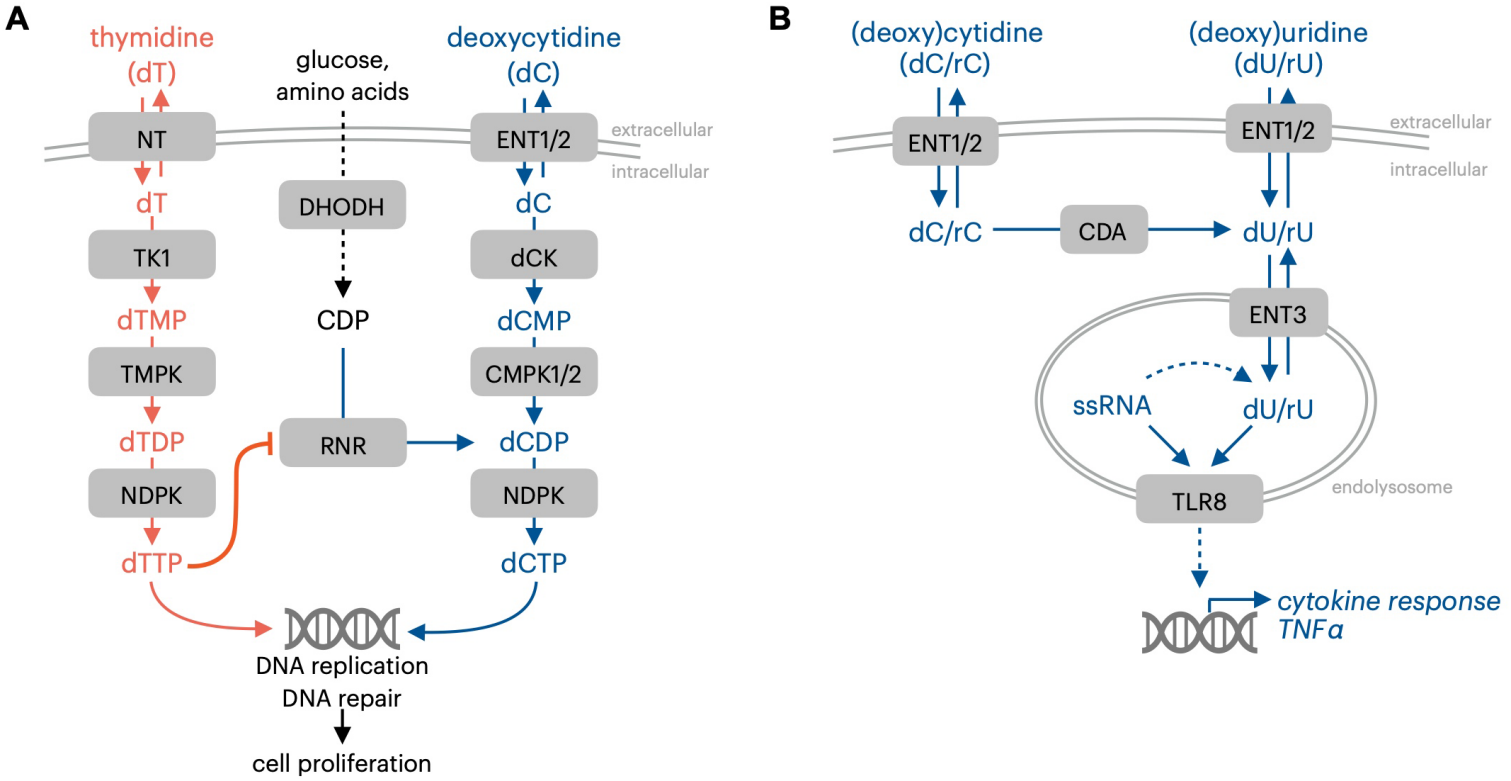
Immune-regulatory effects of pyrimidine nucleosides. **(A)** dCK and dC prevent thymidine-mediated proliferation inhibition resulting from impaired dCTP synthesis. **(B)** Pyrimidine nucleosides are TLR8 ligands. NT, nucleoside transporter TK1, thymidine kinase 1; RNR, ribonucleotide reductase; NDPK, nucleotide diphosphate kinase; ENT1/2, equilibrative nucleoside transporter 1/2 (SLC29A1/2); dCK, deoxycytidine kinase; TMPK, thymidine monophosphate kinase; CMPK1/2, cytidine monophosphate kinase 1/2; CDA,cytidine deaminase; ENT3, equilibrative nucleoside transporter 3 (SLC29A3); TLR8, toll-like receptor 8; ssRNA, single-stranded RNA; rC, cytidine; dC, deoxycytidine; rU, uridine; dU, deoxyuridine.

T cell activation is accompanied by the upregulation of multiple genes in the pyrimidine salvage pathway, including nucleoside transporters and the deoxyribonucleoside kinase dCK. This observation prompted the development of approaches that leverage enhanced nucleoside salvage pathway function in activated lymphocytes to non-invasively track immune responses. Radu and colleagues developed [^18^F]FAC, a pyrimidine deoxyribonucleoside-analog positron emission tomography (PET) probe, to visualize dCK activity as a surrogate marker for immune activation in preclinical mouse models and in humans (35). Following administration, deoxycytidine analog PET probes are transported into cells via nucleoside transporter proteins and are phosphorylated by the pyrimidine deoxyribonucleoside kinase dCK, which effectively traps the probe within cells with elevated dCK activity (35). The biodistribution of deoxycytidine-analog PET probes in preclinical models or in humans can be tracked using a PET scanner. Early studies testing deoxycytidine-analog PET probes in mouse models revealed a striking concentration of pyrimidine deoxyribonucleoside salvage in lymphoid tissues such as the spleen, lymph nodes, thymus, and bone marrow (35). This finding indicated enhanced pyrimidine salvage activity in immune cells *in vivo*. Prompted by this observation, a series of studies in engineered mouse models were performed to evaluate the functional role of dCK by evaluating immune phenotypes in mice where dCK was deleted. These studies provided evidence critically linking dCK function to hematopoiesis and lymphocyte proliferation. The analysis of dCK knockout mice highlighted a requirement for dCK in the development of multiple immune cell lineages, including CD4/CD8 T cells in the thymus, B cells, and erythrocytes (36). The requirement for dCK-mediated dC salvage in normal murine hematopoiesis was, in part, traced to a requirement for dCK to counteract the toxicity resulting from high levels of thymidine in specific tissues (**Figure 2A**) (37). Thymidine is phosphorylated and trapped in cells by thymidine kinase 1 (TK1), and high levels of environmental thymidine drive the expansion of intracellular thymidine triphosphate (dTTP) nucleotide pools. The unbalanced expansion of dTTP pools inhibits dCDP synthesis by RNR, which results in the depletion of dCTP and replication stress in the S-phase of the cell cycle. dCK-mediated dC salvage bypasses this metabolic block to enable proliferation under conditions of high environmental thymidine (37). Preventing thymidine salvage and dTTP pool expansion in dCK knockout mice by inhibiting TK1 prevents replication stress in thymocytes and restores T cell development (37). This data suggests that, in mouse models, dC and dT have major roles in immune cell development and proliferation by functioning as substrates of nucleoside salvage kinases. Similar to the consequences of elevated guanosine nucleoside abundance in PNP deficiency, elevated levels of dT nucleosides restrict hematopoiesis by cell-autonomous lethality. In contrast, dC itself does not appear to exert deleterious effects in hematopoiesis.

In addition to their role as substrates for the nucleoside kinases, nucleosides can be broken down and the resulting ribose can substitute for glucose under conditions of nutrient scarcity. Pancreatic ductal adenocarcinoma (PDAC) cells are resilient, resistant to treatment, and able to thrive in hostile environments by utilizing the pyrimidine nucleoside uridine (180). In low-glucose conditions, uridine phosphorylase 1 (UPP1) is over-expressed, driving the use of uridine as a carbon source that supports macromolecule synthesis and energy generation (180). In cancer cells, UPP1 is controlled by oncogenic KRAS-MAPK signaling and induced by nutrient restriction. Similarly, other nucleosides have been shown to serve as alternative carbon sources, including inosine in CD8 T cells (181) and dT in cancer cells (182).

Altered *de novo* pyrimidine synthesis in humans is associated with immune alterations in the context of the disorder Hereditary Orotic Aciduria (HOA) (38, 39). This rare condition results in defective pyrimidine nucleotide synthesis and is the only identified enzyme deficiency of the *de novo* pyrimidine biosynthetic pathway in humans. HOA is linked to mutational inactivation of uridine-5-monophosphate synthase (UMPS), which leads to decreased pyrimidine synthesis and increased excretion of orotic acid (39, 40). HOA was described as early as 1959 in an infant who passed away before a full investigation could be performed (41). Clinical evidence supports the notion that impaired pyrimidine synthesis results in immunodeficiency, suggesting that pyrimidine nucleotides have immune-strengthening effects. HOA is associated with weakened T cell responses, while humoral responses remain undamaged (38). Symptoms vary across HOA cases but have been reported to include megaloblastic anemia, a weakened immune system, delays in development, and failure to thrive (39). Some symptoms can be treated by supplementation with the pyrimidine nucleoside uridine administered as uridine triacetate.

Pyrimidine nucleosides have roles as metabolic precursors for nucleotide synthesis, and as direct signaling mediators. The endolysosomal pattern recognition receptor Toll-like receptor 8 (TLR8) mediates the signaling effects of pyrimidine nucleosides and possesses a binding pocket that accepts uridine (**Figure 2B**) (42). The nucleoside and oligonucleotide ligands for TLR8 are produced within the lysosomal compartment via RNA breakdown (43). Cytidine and deoxycytidine can be converted to uracil-containing nucleoside TLR8 ligands by cytidine deaminase (CDA) (44). The accumulation of nucleosides within endolysosomal compartments, where they are sensed by TLR7 or TLR8, is mediated by their transport across the endolysosomal membrane by equilibrative nucleoside transporter 3 (ENT3, encoded by the gene SLC29A3). Aberrant endolysosomal TLR signaling resulting from defective SLC29A3-mediated nucleoside transport is linked to H syndrome, an auto-inflammatory condition in humans (45).

Defective activity of the genes controlling the breakdown of pyrimidine nucleosides or nucleobases is linked to the development of various human pathologies. Dihydropyrimidine dehydrogenase (DPYD) catalyzes the first step of uracil and thymine degradation (46). DYPD deficiency (DPD) is a rare metabolic disorder resulting in seizures, developmental delay, microcephaly, and muscular hypotonia, although some patients who are carriers are asymptomatic (47). The activity of other genes and potential environmental factors likely dictate the severity of the manifestations of DPD, causing some individuals to be asymptomatic while others have life-altering manifestations (47). DPD is observed in approximately 3-5% of the population, but the prevalence and phenotypic manifestations vary across ethnic groups (48). DPYD is responsible for the catabolism of 80% of bodily 5-Fluorouracil, a chemotherapy widely used for cancer treatment (49). Therefore, 5-Fluorouracil cannot be used as a cancer treatment for those with DPD, as drug accumulation leads to toxic depletion of pyrimidine nucleotides in this patient population (46).

Mitochondrial neurogastrointestinal encephalomyopathy (MNGIE) is linked to heritable loss-of-function mutations in the gene encoding for thymidine phosphorylase (TYMP). TYMP is a pyrimidine nucleoside phosphorylase that regulates pyrimidine nucleoside salvage by controlling the breakdown of thymidine and deoxyuridine. MNGIE is linked to altered systemic pyrimidine nucleoside accumulation, large-scale disruption of nucleoside metabolism, alongside halted cholesterol, and fatty acid breakdown (50). TYMP deficiency in humans results in the accumulation of thymidine and deoxyuridine, which results in imbalanced nucleotide pools within mitochondria, disruption of mitochondrial DNA replication, and increased mutations. The resulting altered mitochondrial function underlies the manifestations of MNGIE, which include eye muscle weakness, muscle wasting, leukoencephalopathy, digestive dysmotility, microangiopathy, and occasional psychiatric symptoms (50–52). TYMP dysfunction is linked to nucleoside accumulation within lysosomes and the disruption of lysosomal transport proteins. However, it is not clear if any of the manifestations of TYMP deficiency are associated with altered endolysosomal nucleoside sensing by TLR7 or TLR8 (53). Mitochondrial pyrimidine nucleotide imbalance is also linked to the production of immuno-stimulatory type I interferon by triggering release of mitochondrial DNA to the cytosol and downstream cGAS/STING pathway activation (183). The mitochondrial protease YME1L preserves mitochondrial nucleotide pools by preventing pyrimidine nucleotide release to the cytosol via degradation of the mitochondrial nucleotide carrier SLC25A33.

## 4 Immune-regulatory functions of adenosine nucleosides

The nucleoside adenosine is a potent regulator of anti-tumor immune responses that weakens beneficial immune cell subsets and strengthens suppressive cell populations. The role of adenosine in immune system regulation is multifaceted and linked to its role as a signaling molecule and metabolic precursor. Tumor cells co-opt the immunosuppressive effects of adenosine to dampen immune responses by up-regulating the key metabolic enzymes responsible for its production. Therapies that block adenosine generation or sensing have emerged as promising therapeutic targets to reverse immunosuppression in the tumor microenvironment. The mechanisms underlying the effects of adenosine on immune system function and the landscape of therapies targeting the adenosine pathway have been reviewed (1).

Adenosine deaminase activity in humans is mediated by enzymes ADA1 and ADA2. Deficiency in ADA1 results in SCID, while patients with deficiency in ADA2 (DADA2) exhibit a variable clinical phenotype, including systemic inflammation, vasculopathy/vasculitis, and aplastic anemia, with dysregulation of immune, neural, and cardiovascular systems (54, 55). ADA1 does not compensate for dampened ADA2 activity in DADA2 patients. This difference in phenotype resulting from ADA1 or ADA2 deficiency is multifaceted and is linked to differential binding of soluble ADA1 or ADA2 proteins to immune cells (56).

One mechanism by which extracellular adenosine exerts immune-modifying effects is by activating specialized cell-surface receptors which control cell fate and function that are expressed across immune cell lineages. Multiple cell surface receptors for adenosine (A1, A2_A_, A2_B_, and A3) have been characterized (57). A2_A_ is expressed across immune cell types and is well-studied for its role in mediating the immunosuppressive effects of adenosine in the context of anti-cancer immunity. Signaling downstream of A2_A_ is known to exert pleiotropic immune-suppressive functions across immune lineages present in the tumor microenvironment (58, 59). The signaling effects of adenosine have been harnessed for therapy, and synthetic antagonists of adenosine receptors such as vipadenant (BIIB-014) and ST-1535 have shown signs of efficacy in clinical trials for Parkinson’s disease and other conditions (60, 61). Istradefyline, an A2_A_ antagonist, is approved in Japan for Parkinson’s treatment (62). Adenosine signaling via A2_A_ promotes the production of inflammatory cytokines and sustains inflammasome activation following initial activation (63).

The adenosine receptor A2_B_ is also over-expressed in specific cancers (64). It is a low-affinity adenosine receptor compared to A2_A_ and is activated in conditions of high environmental adenosine. It regulates the function of various cell types, including immune and stromal cells, and its inhibition suppresses the growth of tumors in mice (65). The small molecule A2_B_ inhibitor PSB1115 blocks cytokine signaling in stromal cells to limit tumor growth in mouse models (64).

The production of extracellular adenosine in the tumor microenvironment is linked to poor patient outcomes and is driven by high expression of the ectonucleotidases CD39 (ENTPD1) and CD73 (NT5E) (57). CD39 generates AMP from ATP, and CD73 converts AMP into adenosine (66). The expression of adenosine-generating ectonucleotidases is positively regulated by hypoxia and inflammation in the tumor environment (57). The collective preclinical data suggests that therapies blocking CD39 and CD73 could help decrease adenosine production in tumors, thereby unleashing anti-cancer immune responses. This treatment strategy is supported by research testing the consequences of CD39 and CD73 inhibition in preclinical cancer models (57). Prostatic acid phosphatase (PAP) generates adenosine via the breakdown of AMP and may be responsible for immunosuppressive adenosine signaling in prostate cancer tumors via a metabolic pathway that bypasses CD73 (67).

Several approaches for CD73 inhibition are under clinical evaluation as strategies for immunotherapy in patients with solid tumors. Both monoclonal antibodies (68) and small molecule therapeutics (69) that block CD73 activity elicit anti-tumor immune responses and restrain metastasis in murine cancer models. Oleclumab is a CD73-targeting antibody currently under clinical investigation in multiple phase I and II trials and has exhibited promising signs of anti-tumor efficacy (70). Other CD73-targeting antibodies, CPI-006, SRF373/NZV930, and BMS-986179, as well as CD73-targeting small molecules, such as quemliclustat (AB680), are also under clinical evaluation (57).

The anti-cancer effects of preventing adenosine generation using CD39 blocking antibodies have been evaluated in murine models with success (71). POM-1 has been proven as an effective small-molecule CD39 inhibitor that increases cytotoxic T and NK cell activity (72). CD39 is emerging as a therapeutic target to induce anti-cancer immune responses, whereas CD73 and adenosine receptor inhibitors have a more substantial history of research focus (73). Nevertheless, multiple CD39 antagonistic antibodies are undergoing clinical investigation: AB598, TTX-030, IPH5201, and SRF-617, with more in development (74).

Ecto-nucleotide pyrophosphatase/phosphodiesterase (ENPP1) is an additional extracellular enzyme of interest in relationship to adenosine signaling as it degrades purine nucleotides, promoting adenosine production. ENPP1 is connected to the up-regulation of immunosuppressive adenosine signaling and is involved in the breakdown of ATP to AMP via a mechanism that parallels the activity of CD39 (75). ENPP1 also produces AMP via the hydrolysis of the cyclic dinucleotide immuno-transmitter 2’-3’-cGAMP, produced by the enzyme cGAS (76). Potent small-molecule ENPP1 inhibitors such as STF-1623/CM-3163 and AVA-NP-695 are being developed with the potential for cancer therapy (77, 78). Antibodies that target ENPP1 to prevent its enzymatic activity have been developed and tested in preclinical models of myocardial infarction to limit the cell death and fibrosis that is linked to increased ENPP1 activity following cardiac injury (79, 80).

Adenosine can be produced in the extracellular environment by the breakdown of nucleotides released by dying cells via CD39, ENPP1, and CD73. It is also released by live cells via equilibrative nucleoside transporters. Inhibition of nucleoside transport is currently under investigation as an alternative approach to ectonucleotidase inhibition to limit immunosuppressive adenosine signaling for cancer immunotherapy (81).

The purine nucleoside inosine, a product of adenosine deamination via ADA, has also been linked to cancer progression and metastasis, acting as a precursor for nucleotide synthesis in the tumor microenvironment during starvation (82, 83). In addition, inosine regulates the phenotype of T cells (84). One emerging tactic to leverage the immune-modifying properties of inosine is to improve CAR-T therapy by over-expressing ADA to promote the conversion of immunosuppressive adenosine to inosine. This approach increases the functionality and stem cell-like properties of CAR-T cells, which amplifies their anti-cancer effects (84).

The systemic inflammatory condition known as Still’s disease is spurred by genetic loss-of-function mutations in the gene FAMIN, which encodes an enzyme with a roles in adenosine, guanosine and inosine metabolism as well as the prevention of autoimmunity and pathogenic T cell activation. Still’s disease manifests in childhood, with recurrent fevers and arthritis being the most common phenotypes, although 20% of those with the condition develop macrophage activation syndrome. The enzymatic function of FAMIN overlaps with that of ADA, PNP and MTAP. Compromised FAMIN function in dendritic cells is linked to aberrant NAD/NADH metabolism antigen presentation, and inosine metabolism that together contribute to enhanced T cell priming (179).

## 5 Roles of nucleoside transporters in immune regulation

Systemic nucleoside abundance is tightly controlled by proteins that regulate nucleoside production, breakdown, and translocation across plasma membranes (85). Nucleoside uptake and release in live cells is mediated by specialized multi-pass transmembrane transporter proteins (85). In addition to their role in controlling the systemic levels of nucleosides, nucleoside transporters enable the nucleoside salvage pathway for nucleotide synthesis in cells. Equilibrative nucleoside transporters 1 and 2 (ENT1/2, encoded by the genes SLC29A1/2) mediate the passive transport of nucleosides across the plasma membrane along a concentration gradient, whereas concentrative nucleoside transporters CNT1 and CNT2 (encoded by the genes SLC28A1/2) mediate the sodium-coupled secondary active transport of nucleosides (86). The ENT family member SLC29A3 (ENT3) is located on the lysosomal membrane within cells and mediates the transfer of nucleosides across intracellular compartments (87). SLC29A4 (ENT4) functions as a plasma membrane polyamine transporter (88, 89).

Nucleoside transporters accept various substrates, including natural pyrimidine and purine nucleosides, synthetic anti-metabolite nucleoside analogs (such as gemcitabine, cytarabine, and clofarabine), and radionuclide-labeled nucleoside-analog PET imaging probes (such as [^18^F]FAC) (85). Cancer cells lacking nucleoside transporter activity are resistant to nucleoside-analog prodrugs (90). SLC29A1 (ENT1) is the predominantly expressed nucleoside transporter across normal and tumor cells and facilitates the utilization of environmental nucleosides for nucleotide synthesis. Beyond their ability to provide metabolic precursors to cells, nucleoside transporters also control the access of nucleosides to their sensors (such as adenosine receptors, TLR7, and TLR8). ENT1 mutations have been identified in human patients. The manifestations of impaired ENT1 activity in humans include ectopic mineralization, joint calcification, and dysregulated erythropoiesis (94, 95). The Augustine blood group system includes antigens encoded by various SLC29A1 alleles (96).

ENT1 mediates the immune-regulatory effects of adenosine in part by controlling its uptake in lymphocytes. Adenosine uptake is linked to pyrimidine synthesis inhibition via phosphoribosyl pyrophosphate synthetase (PRPS) and a resulting proliferation block in tumor-infiltrating T cells (91). Therefore, pharmacological ENT1 inhibition has been suggested as a strategy to enhance anti-cancer T cell responses and ENT inhibitors have emerged as a rational companion therapy for immune checkpoint blockade. Nucleoside transporters also have a critical role in dictating the immunological outcomes driven by guanosine nucleosides as their transport across the plasma membrane is mediated by ENTs (92, 93). The anti-proliferative effects of guanosine supplementation in culture are curbed by ENT1 inhibition.

Inactivation of lysosomal membrane nucleoside transport resulting from mutations in ENT3 is associated with hyperactive immune phenotypes in humans with genetically inherited disorders (97). ENT3 controls the abundance of guanosine and uridine nucleosides within lysosomes, which function as ligands for TLR7 and TLR8, respectively. Lymphocytes can use ENT3-mediated nucleoside recycling to support nucleic acid synthesis and sustain proliferation (97). ENT3 deficiency results in dysregulated nucleoside transport across lysosomal membranes, leading to nucleotide pool imbalance, metabolic stress, and aberrant TLR-driven cytokine responses (45). Germline loss-of-function SLC29A3 (encoding for ENT3) mutations in humans are notably associated with irregular histiocyte production and accumulation, causing autoimmune responses presenting as the genetic disorder H syndrome (98). Cases of H syndrome are rare, with patients presenting with pigmented hypertrichosis with insulin-dependent diabetes mellitus (PHID), Faisalabad histiocytosis, and sinus histiocytosis with massive lymphadenopathy (45, 97, 99, 100). These phenotypes are potentially linked to aberrant macrophage activation and accumulation in the spleen and other organs due to nucleoside accumulation in lysosomes and subsequent TLR7 or TLR8 activation (45, 101). Interestingly, while the manifestations of ENT3 deficiency in mice appear to be driven by aberrant TLR7 signaling, the consequences of ENT3 deficiency in human-derived cells are mediated by TLR8 (45).

## 6 Emerging therapeutic strategies to unleash the immune-stimulatory effects of nucleosides

### 6.1 PNP inhibition

Decades after the initial observations of T cell insufficiency in patients with ADA or PNP-linked SCID by Giblett and colleagues, their discovery was leveraged for therapy. Low PNP activity in humans is associated with decreased T cell counts, making T cell malignancies a natural place to examine the benefit of pharmacological PNP inhibition (102). PNP inhibition was hypothesized to selectively elevate deoxyguanosine levels in malignant T cells, leading to dGTP accumulation and cancer cell death (102, 103). In the 1990s, Schramm and colleagues applied their knowledge of the PNP transition state substrate-enzyme structure to design PNP inhibitors with exceptionally high potency (104, 105) (**Table 1**). Pharmacological PNP inhibition was found to selectively eradicate T cell leukemia cells *in vitro*, thus mirroring the observations of T cell deficiency in patients with PNP-linked SCID (105). These encouraging preclinical results prompted the testing of the PNP inhibitor forodesine (also known as BCX-1777 or Immucillin H) in clinical trials for relapsed/refractory T and B cell leukemias and lymphomas (106). Forodesine received approval for treating peripheral T cell lymphoma in Japan in 2017 (102, 107). Despite excellent tolerability and pharmacodynamic properties in humans, evidenced by plasma accumulation of PNP substrates and depletion of the downstream products of PNP (including uric acid), durable responses were observed only in a subset of patients. Additional PNP inhibitors, ulodesine and peldesine, with potency and bioavailability comparable to forodesine, have entered clinical trials for applications beyond cancer treatment, such as arthritis or limiting uric acid accumulation in gout (108). It has been noted that PNP inhibitors have lower efficacy against cancer cells *in vivo* compared to cell culture experimentation. This discrepancy may be due to the presence or absence of factors not accounted for in cell culture models or specific genetic differences of the cancer cells targeted in each study (106).

**Table 1:** Landscape of therapeutic targets for immune modulation within nucleoside metabolism.

Target	Notable Therapeutic Agents	Notes on Function and References
PNP	-Forodesine (106) -Ulodesine (108) -Peldesine (108)	-PNP inhibitors stabilize purine nucleosides (16) -PNP inhibitors promote germinal center reactions by stabilizing the TLR7 ligand guanosine (10) -Forodesine is approved for the treatment of T Cell lymphoma (Japan) (102, 107) -PNP inhibition is synthetic lethal with SAMHD1 inactivation (10, 109)
DHODH	-Leflunomide (138) -Teriflunomide (138)	-DHODH inhibitors prevent de novo pyrimidine synthesis (138) -Lefunomide is approved for the treatment of rheumatoid arthritis and psoriatic arthritis (138) -Teriflunomide is approved for the treatment of patients with relapsing forms of multiple sclerosis (138) -DHODH inhibition enhances tumor cell antigen presentation and response to immune checkpoint blockade (144) -DHODH inhibitors tune the developmental trajectory of immunization-elicited T cells elicited from predominantly short-lived effectors to a memory phenotype (145)
SLC29A1 (ENT1)	-Dipyridamole (124) -NBMPR (128) -Dilazep (128)	-ENT inhibitors block nucleoside uptake in cells and cause systemic nucleoside accumulation (81) -Blocking ENT1-mediated adenosine uptake in T cells enhances anti-tumor immunity in mouse models (91)
dCK	-TRE-515 (119)	-dCK inhibitors block the salvage of intact pyrimidine deoxyribonucleoside dC, dA and dG (10, 37) -dCK inhibition is effective for the treatment of multiple sclerosis in mouse models (184, 185) -TRE-515 is under clinical investigation for the treatment of solid tumors (NCT05055609) (119)
CD73 (NT5E)	-AB680 (57) -Oleclumab (70)	-CD73 inhibition prevents the production of immunosuppressive adenosine (57, 60) -CD73 inhibition is currently under clinical investigation for the treatment of solid cancers in combination with chemotherapy (70)
CD39 (ENTPD1)	-AB598 (74) -TTX-030 (74) -IPH5201 (74) -SRF-617 (74)	-CD39 inhibition stabilizes ATP and prevents its conversion to immunosuppressive adenosine (57, 66) -CD39 inhibition is currently under clinical investigation for the treatment of solid tumors (74)
ENPP1	-AG-3132/AG-3292 (77) -AVA-NP-695 (78)	-ENPP1 inhibitors prevent the breakdown of immuno-stimulatory 2'-3'-cGAMP and the resulting production of adenosine (76) -Currently advancing towards clinical trials for the treatment of solid cancers (77, 78)

PNP inhibitors appear to be most effective in inducing apoptosis in cancer cells deficient in the dNTP triphosphohydrolase SAMHD1 (10, 109). SAMHD1 degrades dNTPs to their corresponding nucleosides and prevents the expansion of intracellular dNTP pools (110). When challenged with PNP inhibitors, human and mouse cells without SAMHD1 are eradicated, while cells expressing SAMHD1 survive, indicating that SAMHD1 and PNP are a pair of synthetic lethal genes (109). SAMHD1 has, therefore, emerged as a crucial biomarker for the anti-cancer effects of PNP inhibitors. This insight has increased the potential clinical utility of PNP inhibitors by providing the rationale for treating solid tumors with low SAMHD1 expression (105). Loss-of-function SAMHD1 mutations occur in several cancer types, including lymphocytic leukemia, lung, and colon cancer (105, 111). SAMHD1 expression is controlled in part by transcriptional upregulation downstream of signaling driven by the cytokine interferon (112). PNP is up-regulated in certain cancers, such as pancreatic adenocarcinoma, where it may be a therapeutic target (113).

While PNP inhibitors were initially applied to eradicate malignant lymphocytes, immune-activating effects were noted in patients receiving this new type of treatment. These effects included enhanced responses to vaccines and beneficial effects in the context of post-HSCT relapse in patients with leukemia (114). Based on these observations, it was hypothesized that the immune stimulatory properties associated with PNP inhibitor treatment may result from the activation of immune sensor molecules such as TLRs. Consistent with this model, recent studies have indicated that the PNP substrates guanosine and deoxyguanosine activate TLR7.

Oral treatment with PNP inhibitors triggers transcriptional alterations in B cells, dendritic cells, and macrophages via TLR7 activation (10, 24). The transcriptional alterations in macrophages driven by PNP inhibitors, resulting from an accumulation of the endogenous TLR7 ligand guanosine, are distinct from those elicited by synthetic guanosine-analog TLR7 agonists, such as R848. An advantage for PNP inhibitors over synthetic agonists for therapeutic TLR7 agonism is the difference in the duration and magnitude of cytokine responses elicited by either therapy. While synthetic TLR7 agonists trigger an acute, transient high-level of TLR7 activation, PNP inhibitors may induce a lower level of activation but a long-lived response resulting from the sustained systemic accumulation of the purine nucleoside TLR7 ligands. Targeting the PNP-regulated immune checkpoint in patients may enhance anti-tumor immune responses or vaccine-driven humoral and T cell responses.

### 6.2 Nucleoside salvage kinase inhibition

The development of selective, potent, and orally bioavailable dCK inhibitors was guided by structural analysis and preclinical imaging studies that leveraged dCK-specific PET probes (115–119). The dCK inhibitor DI-87 (TRE-515) was developed to eradicate pathogenic cell types that rely on dCK activity (119). dCK inhibitors are well-tolerated in preclinical models and have minimal effects on normal cells. The first-in-class dCK inhibitor TRE-515 is currently under clinical investigation for the treatment of solid tumors (NCT05055609).

dCK inhibition is a promising anti-cancer treatment strategy, as tumor cells exhibit an increased demand for pyrimidine nucleotide synthesis to fuel DNA replication and repair. dCK inhibitors trigger replication stress alongside lethal DNA damage in tumor cells, and improve survival in mouse models of acute lymphoblastic leukemia (ALL) when administered alongside inhibitors of *de novo* dCTP synthesis, such as thymidine or triapine (117, 120). As a mono-therapy, dCK inhibitors may be most effective for treating specific tumors that exhibit a diminished capacity for *de novo* pyrimidine nucleotide synthesis resulting from transcriptional suppression, mutational inactivation, or nutrient scarcity (119).

Based on their strong safety profile and unique mechanism of action, dCK inhibitors are a promising companion for established treatments that induce DNA damage or restrict *de novo* pathway activity in tumor cells. dCK mediates radiation resistance by supplying the pyrimidine dNTP precursors needed for DNA repair (121). dCK inhibitors also have potent anti-cancer effects against cells deficient for the tumor suppressor gene BRCA2 (122). Therefore, dCK inhibitors are potentially a high-priority companion therapy for PARP inhibitors for this genetically-defined cancer type.

Preclinical observations of altered immune phenotypes in dCK knockout mice prompted the testing of a dCK inhibitor for treating autoimmune diseases. In this setting, dCK inhibitors may block a selective requirement of disease-driving lymphocyte populations on enhanced dCK activity while sparing normal cells that utilize the *de novo* pathway to satisfy their dNTP requirements. dCK inhibitors have demonstrated the potential to mitigate the manifestations of multiple sclerosis in mouse models, and dCK-specific PET probe accumulation has been proposed as a potential non-invasive biomarker for these inhibitors in patients (37, 123, 184, 185). Based on promising preclinical data, dCK inhibitors are progressing toward clinical development to curtail aberrant immune activation.

### 6.3 Nucleoside transport inhibition

Nucleoside transport across the plasma membrane is a critical step for nucleotide synthesis via salvage pathways and controlling the nucleoside levels in the extracellular environment. ENT inhibition is currently under evaluation as an approach to limit immunosuppressive adenosine signaling in tumors (81). Targeting ENT1 may enhance T cell-mediated tumor cell killing by (i) limiting the release of adenosine by tumor cells to prevent adenosine receptor signaling, and (ii) blocking the anti-proliferative effects resulting from adenosine uptake in immune cells. FDA-approved dipyridamole inhibits ENT1, effectively preventing adenosine uptake, particularly across inflammatory states with excessive adenosine production (124). The immune-stimulatory and anti-cancer effects of nucleoside transporter inhibition are also linked to the protection of tumor-infiltrating lymphocytes by preventing the pyrimidine *de novo* synthesis pathway defect triggered by excessive adenosine salvage (91).

Dipyridamole has cellular targets beyond ENT1. Therefore, its clinical utility in cancer immunotherapy is limited. NBMPR is a potent ENT1 inhibitor but has not been used directly as an anti-cancer therapeutic (127). The crystal structures of ENT1 in complex with two established inhibitors of adenosine re-uptake, NBMPR and Dilazep, have been solved (128), and this information may guide the development of new ENT inhibitors with improved target engagement and specificity that are suitable for clinical use. Nucleoside transport inhibitors may have anti-cancer effects when applied as a mono-therapy (125) and can potentially prevent resistance to DNA-damaging chemotherapeutics by limiting the synthesis of nucleotides via the salvage pathway that may support DNA repair (126).

### 6.4 TYMP inhibition

The link between MNGIE and altered TYMP activity prompted an investigation into the mechanisms linking thymidine metabolism to mitochondrial function in other contexts, such as cancer. TYMP up-regulation is associated with pro-tumor functions such as cancer cell proliferation, metabolic alterations, and increased angiogenesis (129). Many cancers utilize TYMP-mediated pathways to form 2-deoxyribose that can fuel biosynthetic processes (129). Therefore, blocking TYMP represents a potential anti-cancer treatment strategy. The TYMP inhibitor tipiracil hydrochloride (TPI) restrains basement membrane incursion to prevent metastasis and trigger apoptosis (130). In addition to their role in inhibiting pyrimidine salvage, TYMP inhibitors may have utility for restraining the production of the TLR8 ligand deoxyuridine from thymidine to limit uncontrolled immune responses.

### 6.5 MTAP inhibition

5’-methylthioadenosine (MTA) phosphorylase (MTAP) is an enzyme with a role in the metabolism of polyamine as well as the salvage pathway for the synthesis of adenine and methionine (131). MTAP degrades MTA into S-adenosyl-L-methionine (SAM) (132). The deleted form of the MTAP gene occurs in approximately 15% of cancers and has been linked to immune evasion (133, 134). MTDIA (Methylthio-DADMe-Immucillin-A) is an MTAP inhibitory molecule (133). In mouse models of lung and colorectal cancer, MTDIA therapy exhibited considerable anti-tumor effects, extending survival and reducing tumor growth (132, 135, 136). Unlike many other therapies, there is little high-dosage toxicity with MTDIA treatment, indicating that this therapy is suitable for extended use (132). When MTDIA is not administered, MTAP metabolizes MTA into adenosine and 5-methylthioribose-1-phosphate (MTR-1-P), allowing for cancer cell proliferation. When MTDIA is administered and MTAP is inhibited, PRMT5-mediated histone methylation and intron splicing are competitively decreased, resulting in the restraint of cancer growth (132).

### 6.6 DHODH inhibition

The increased requirement of activated lymphocytes on nucleotide synthesis has been leveraged therapeutically, as the inhibition of *de novo* pyrimidine nucleotide synthesis is an established treatment strategy for the management of autoimmune disorders (137). Inhibition of *de novo* pyrimidine synthesis using dihydroorotate dehydrogenase (DHODH) inhibitors is an FDA-approved approach to combat multiple sclerosis and rheumatoid arthritis (138). The DHODH inhibitor Leflunomide was approved in 1998 for treating rheumatoid arthritis. This was followed by the approval of Teriflunomide for multiple sclerosis in 2012. In these autoimmune disorders, pyrimidine synthesis-targeting drugs are administered to prevent the aberrant proliferation of immune cells that drive the autoimmune manifestations (139).

There is potential for DHODH inhibitors in cancer treatment, as DHODH has a central role in sustaining cancer cell proliferation and regulating anti-cancer immune activity. DHODH inhibition using small molecule drugs is effective for the treatment of preclinical cancer models such as small cell lung cancer (140), MYC-amplified medulloblastoma (141), and IDH1 mutant glioma (142). In addition to promoting nucleotide synthesis, DHODH activity strengthens cancer cells by providing defense against ferroptosis (143). DHODH inhibitors also reprogram myeloid differentiation, and this effect may be relevant for treating myeloid leukemias (137).

In mouse models, DHODH inhibition enhances the efficacy of immune checkpoint blockade using anti-CTLA-4 with anti-PD-1 antibodies by up-regulating the expression of antigen presentation pathway genes in cancer cells (144). The modulation of pyrimidine nucleotide synthesis using DHODH inhibitors also impacts T cells directly and has been shown to tune the developmental trajectory of immunization-elicited T cells elicited from predominantly short-lived effectors to a memory phenotype (145). The impact of nucleoside transport or salvage pathway inhibition on this process has yet to be defined.

### 6.7 Modified nucleoside therapies

While the structural basis for the sensing of guanosine by TLR7 has only recently been described, the immuno-stimulatory effect of small molecule guanosine analogs has been known for decades (146, 147). Guanosine analogs have immuno-stimulatory properties via the activation of TLR7, and guanosine-analog TLR7 agonists have been evaluated as a form of cancer immunotherapy (147). TLR7 activation by synthetic guanosine analogs bypasses the requirement for TLR7 binding to ssRNA. Guanosine derivatives, such as Loxoribine, have been developed as therapeutic agents to activate TLR7. This class of agonists initiate intracellular signaling cascade involving proteins such as p50 and p65, which drive the expression of pro-inflammatory cytokines (148, 149).

## 7 Lost in translation: differences between mouse and human metabolism is a significant obstacle in the preclinical study of the immune-regulatory functions of nucleosides

The disparities between mouse and human nucleoside metabolism limit the translational impact of the promising results obtained from experiments that use mouse models (150). These significant differences may produce confounding results and hinder the translation of new therapeutics. For example, pyrimidine deoxyribonucleoside concentrations are measured at levels that are orders of magnitude higher in rodent plasma than in humans (2, 151, 152). The variation in the systemic levels of nucleosides across species is related to differences in the expression and activity of enzymes involved in nucleoside breakdown. Distinct diet and behavioral patterns may also contribute to these differences.

The discrepancy in the expression and activity of nucleoside catabolism-related genes across species is a central contributor to the differences in the measured levels of systemic nucleosides. Mice are deficient for the enzyme ADA2 (encoded by the gene CECR1), which catalyzes the conversion of (deoxy)adenosine to (deoxy)inosine and has a ~100-fold lower affinity for free adenosine nucleosides than ADA1. ADA2 is broadly expressed in human cell types and is reported to function within endolysosomes to regulate TLR9 signaling with DNA as its primary substrate (153, 154). This cross-species metabolic incongruence complicates the extension of findings in mouse models regarding the links between adenosine deamination and immune activation in the human setting. The disconnect between human and mouse models is also highlighted by research involving the adenosine-generating enzyme CD73. CD73 deficiency in humans is associated with calcification of small joints, vascular calcification, and arteriomegaly; in contrast, CD73-deficient mice do not exhibit an apparent phenotype (155).

Studies of the bio-distribution of deoxyribonucleoside-analog PET probes across mice, dogs, non-human primates, and humans reinforce the differences in nucleoside metabolism across species. The thymidine analog PET probe [^18^F]FLT exhibits no specific tissue accumulation pattern in rodent models (156, 157). However, in humans, this probe accumulates in tumors and secondary lymphoid tissues characterized by high levels of cell proliferation and TK1 expression. One factor underlying this difference is differential systemic levels of plasma thymidine concentrations across mice and humans (2). Both thymidine and [^18^F]FLT require transport by plasma membrane transporters and phosphorylation by TK1 for their intracellular trapping. Thymidine competes with [^18^F]FLT for phosphorylation by TK1 as the fluorine substitution significantly decreases its affinity for TK1 (158, 159). One explanation for the difference in thymidine metabolism between mice and humans is the differential expression or activity of the enzyme responsible for thymidine breakdown, TYMP.

Differences in thymidine metabolism between mice and humans complicate the application of mice for MNGIE studies and result in diverging immune responses following TYMP inhibition (152). This discrepancy is due to several factors, including low TYMP levels in murine blood compared to humans, altered nucleoside levels in plasma, and the complementary role of uridine phosphorylase to TYMP in catabolizing dT and dU, providing a biochemical route to degrade these deoxyribonucleosides (152). In mice engineered to be deficient in uridine phosphorylase and TYMP, there is 1/10th the level of dU and dT increase compared to humans (160). This correlates with an incomplete pallet of symptoms in mice, which often lack the hallmark gastrointestinal and muscular manifestations (160). Furthermore, a heightened pyrimidine pool in mice could make specific cancer treatments appear more effective, as depletion of pyrimidines would result in a more drastic decrease in murine models than in humans. Similarly, while mice with PNP deficiency recapitulate the T cell deficiency observed in humans lacking PNP, mice experience a less severe phenotype, often lacking neurological symptoms (10, 161).

A similar challenge was encountered in translating the deoxycytidine-analog PET probes to monitor dCK activity non-invasively *in vivo*. While the first-generation dCK-specific PET probe [^18^F]FAC effectively visualized cell proliferation in lymphoid tissues in mice, it did not exhibit a specific uptake pattern in humans (2, 162). This species-specific tissue accumulation pattern of the dCK-specific PET probes was traced to the differential activity of CDA, the enzyme responsible for deoxycytidine catabolism, across mice and humans (163). Mice exhibit lower CDA activity, which may explain their higher plasma concentrations of pyrimidine nucleosides. This difference could account for variations in pyrimidine analog drug breakdown, as the slower breakdown in rodents is likely due to less active CDA (164). In addition to the natural pyrimidine deoxyribonucleosides, [^18^F]FAC is susceptible to CDA-mediated catabolism. This finding prompted the development of a next-generation dCK-specific PET probe resistant to CDA. [^18^F]CFA is a purine nucleoside analog that requires phosphorylation by dCK for its intracellular trapping but is not a substrate for CDA (2). [^18^F]CFA has shown promise for the noninvasive measurement of dCK activity in humans using PET imaging (2, 165).

Significant disparities also exist between mouse and human immune systems (150). While there are distinct patterns of expression or activity of genes within nucleoside metabolism between mice and humans, PRR-family nucleoside sensors also exhibit species-specific expression patterns. In particular, the uridine nucleoside sensor TLR8 is expressed at low levels in murine cells compared to human cells. This difference may underly the discrepancy in the manifestations of ENT3 deficiency across mice and humans, with humans presenting with auto-inflammation that is not fully recapitulated in SLC29A3 knockout mice (101). Transgenic mice have been developed to recapitulate the expression of TLR8 observed in humans (166). Nevertheless, this difference in PRR expression exemplifies the different biological environments of the two species that need to be considered when performing experiments in preclinical models.

## 8 Challenges and opportunities in the development of model systems to study the links between the human immune response and nucleoside metabolism

Improved preclinical tissue culture systems and mouse models that recapitulate both human nucleoside metabolism and immune responses are needed to facilitate the translation of new metabolism-targeting therapies. Promising advances have been made in engineering new mouse models for human immune responses. Interestingly, these models also recapitulate some aspects of human nucleoside metabolism, and may enable the evaluation of the effects of nucleoside metabolism-targeting therapies on immune system function. One approach for this is “humanized mouse models,” a system where mice are engineered with human tissues to recapitulate components of the human immune system, which is useful to study human tumor conditions and therapy responses (167). These models have been applied to evaluate antibodies, adoptive cell therapies, oncolytic viruses, and small molecule inhibitors (167). Immunodeficient mice are often the hosts for the immune engraftments, and there have been steady improvements to mouse strains and techniques over the last 50 years, allowing for decreased rejection of human cells upon transplantation (168). Multiple murine humanization techniques have been developed, including Hu-PBL, Hu-SRC, and Hu-BLT.

Hu-PBL is a relatively straightforward humanization method that involves the transplantation of human peripheral blood mononuclear cells (PBMC) into immunocompromised murine hosts (168). This engineering technique results in a human immune system mainly composed of T cells, albeit with diminished human cytokine levels and weak propagation of B and NK cells (168). This model is, therefore, best suited to test therapeutics and systems focusing on T cell behavior. A limitation of this model is that it often results in graft-versus-host disease (GVHD), limiting the scope and potential time frame for experiments (167).

The Hu-SRC technique more accurately captures the spectrum of human immune cell types (168). It involves the transfer of CD34+ hematopoietic stem cells (HSCs), which allows for the development of more complex innate and adaptive immune systems. Compared to Hu-PBL, it is a more stable model, with fewer instances of rejection (168). However, it may involve deficiencies of innate cell lineages and reduced B cell functionality (167).

Hu-BLT (bone marrow, liver, thymus) is a complex and more complete immune modeling system. It combines the Hu-SRC protocol of CD34+ hematopoietic stem and progenitor cell (HSPC) injection with particles of the human fetal thymus and fetal liver into immunodeficient mice (168, 169). This results in the growth of a human thymus analog within the mouse (167). However, there is still susceptibility to GVHD and rejection (168). Although certain strains of mice appear to resist rejection, obtaining sufficient human tissue for implantation complicates the engineering of this model (167). Notably, BLT mice recapitulate some aspects of human purine and pyrimidine metabolism, including lower systemic pyrimidine levels and enhanced pyrimidine catabolism (10, 169). BLT humanized mice, or next-generation humanized mice, may provide a powerful foundation for the investigation of new therapies that target nucleoside metabolism for immune modulation.

An alternative system for monitoring the interactions between human nucleoside metabolism and immune responses is the *ex vivo* culture of human tissue. These models involve the culture of primary human cells or explanted human donor material to recapitulate the heterotypic cellular composition of tissues, including tumors. These *ex vivo* models are an emerging platform to evaluate immune-based therapies and may be suitable for studying the immuno-modulatory effects of nucleoside metabolism-targeting therapies.

Patient-derived organoids are an *ex vivo* method for studying individual tumor responses to intervention. This method involves the collection of tissue from a patient, from which cancer cells are isolated and cultured to form 3D organoid structures (170). Organoids have been successfully formed from various tumor types (170). These models can potentially test whether a patient would respond to specific therapy (170).

Precision-cut tumor slices (PCTS) offer an experimental platform to model the intricate *in vivo* tumor environment in cell culture conditions (171). This system involves the culture of thinly sliced human or mouse tissue sections under specialized culture conditions. PCTS maintain integrity for 3–12 days, depending on culture methods and cancer type (171, 172). In contrast to organoid models, PCTS more completely encompass the heterotypic cellular composition of tissues. Several challenges with this model must be considered, including ischemia, hypoxia, loss of integrity during slicing, and the preservation of slices using cell culture methods (173). Multiple reports also suggest significant transcriptional changes in the hours after slices are prepared, and down-regulation cytokine production has been observed (173, 174). These models offer opportunities for developing personalized therapeutic approaches for cancer, as immunotherapies can be specified to the patient after tumor testing (173). The PCTS model has been applied to model immunosuppressive mechanisms operating in the tumor microenvironment and monitor the effects of immune-based anti-cancer therapies (175–178). PCTS models are a promising platform for future investigations of the immune-modifying properties of nucleoside metabolism-targeting therapies.

## 9 Conclusions

Over the past several years, substantial progress has been made in understanding the mechanisms underlying the immune-modifying effects of purine and pyrimidine nucleosides. This advancement was possible due to the commitment of scientists and physicians toward the development of new tools to measure and modify nucleoside metabolism in humans. However, the full therapeutic potential of nucleoside metabolism-targeting interventions for patient care has yet to be fully realized. Results from ongoing clinical trials evaluating the modification of adenosine signaling for cancer immunotherapy will undoubtedly provide new insight that may be applied in future clinical investigations. The development of new preclinical models that recapitulate human nucleoside metabolism is a central obstacle in translating new mechanistic insights from laboratory experiments into therapies. These models may provide insights into the therapeutic contexts and disease types where specific metabolism-targeting therapies will be most effective. New mouse models that possess a humanized nucleoside metabolism and immune system hold immense promise as a platform for these studies. *Ex vivo* cultures of primary human tissues may also serve as a valuable and relevant platform for future investigations of the intersections between nucleoside metabolism and immune system function.
